# Clinical Efficacy and Safety of Nerve-Sparing Radical Hysterectomy for Cervical Cancer: A Systematic Review and Meta-Analysis

**DOI:** 10.1371/journal.pone.0094116

**Published:** 2014-04-18

**Authors:** Ying Long, De-sheng Yao, Xin-wei Pan, Ting-yu Ou

**Affiliations:** 1 Department of Gynecologic Oncology, Affiliated Tumor Hospital of Guang Xi Medical University, Nanning, People's Republic of China; 2 Department of Gynecology, Third Affiliated Hospital of Guang Xi Medical University, Nanning, People's Republic of China; State University of Maringá/Universidade Estadual de Maringá, Brazil

## Abstract

**Backgroud and Objective:**

Nerve-sparing radical hysterectomy (NSRH) may be associated with lower postoperative morbidity than radical hysterectomy (RH). We aimed to compare the clinical efficacy and safety of abdominal or laparoscopic NSRH and RH for treating cervical cancer through systematic review and meta-analysis.

**Methods:**

PubMed, EMBASE, The Cochrane Library and the Chinese National Knowledge Infrastructure databases were systematically searched for all relevant studies. Data were abstracted independently by two reviewers. A meta-analysis was performed to compare intra- and postoperative outcomes for the two techniques.

**Results:**

A total of 17 clinical trials were identified. Meta-analysis showed that although operating time was significantly longer for abdominal or laparoscopic NSRH than for RH, NSRH based on laparotomy or laparoscopy proved more effective for postoperative recovery of bladder function. NSRH was also associated with lower bladder dysfunction morbidity and fewer postoperative complications. Two abdominal trials and one laparoscopic study further suggested that NSRH was associated with shorter time to recovery of anal/rectal function. In contrast, RH and NSRH based on laparotomy or laparoscopy were similar in terms of extent of resection, recurrence rate, survival rate, blood loss and frequency of intraoperative complications. The meta-analysis showed that abdominal NSRH was not significantly different from RH in length of hospital stay, while one trial suggested that length of hospital stay was shorter after laparoscopic NSRH than after the corresponding RH.

**Conclusion:**

NSRH may be a reliable technique for treating early cervical cancer. Available evidence suggests that it is better than RH for postoperative recovery of pelvic organ function and postoperative morbidity, while the two techniques involve similar clinical safety and extent of resection. These results should be considered preliminary since they are based on a relatively small number of controlled trials, most of which were non-randomized. The findings should be verified in larger, well-designed studies.

## Introduction

Conventional surgical management of early-stage cervical carcinoma is radical hysterectomy (RH), which is associated with postoperative morbidities like bladder dysfunction, sexual dysfunction and colorectal motility disorders. Accidental damage to the pelvic autonomic nerves during surgery is thought to be a major cause of these morbidities [1]–[3]. Improving surgical treatment as well as postoperative quality of life are increasingly important challenges given that more than 54% of women diagnosed with cervical cancer are younger than 50 years [4]. As a result of advances in minimally invasive surgery, laparoscopic radical hysterectomy (LRH) is now performed routinely around the world [5]. While this technique is less invasive than RH, it can still lead to substantial rates of postoperative morbidity.

In an effort to reduce postoperative morbidity, many gynecologists have focused on surgical approaches that protect the pelvic nerves that can be damaged during RH. The first approach, called nerve-sparing radical hysterectomy (NSRH), was invented by Japanese gynecologists. NSRH has been adopted and developed over the last 20 years by surgical schools around the world [6]–[8]. More recently, laparoscopic NSRH (LNSRH) has been increasingly applied to operable cervical carcinoma [9]–[11]. Many clinicians believe that the nerve-sparing approach is associated with lower postoperative morbidity than non-nerve sparing RH, with similar clinical efficacy and safety. We decided to test this belief rigorously by conducting a systematic review of the literature and meta-analysis of pooled studies.

## Methods

### Search strategy

All relevant studies published in English and Chinese up to July 30, 2013 were identified through systematic searches in PubMed, EMBASE, the Cochrane Library database and the Chinese National Knowledge Infrastructure (CNKI) database. The search terms used were: nerve sparing, radical hysterectomy, preserve nerve, and all these terms in combination with cervix carcinoma or cervical cancer. Reference lists in all relevant articles were also manually searched.

### Study eligibility

A study was included in the meta-analysis if it involved (1) patients with biopsy-proven cervical cancer, regardless of age, ethnicity or location; (2) a randomized or non-randomized controlled design, or a case-control design; (3) laparotomy or laparoscopy; (4) comparison of clinical efficacy of Type III NSRH with Type III RH; and (5) evaluation of at least one outcome from among the following: operating time, intraoperative blood loss, hospital stay, bladder function recovery, anorectal function recovery, sexual function recovery, intra- and/or postoperative complications, survival rate, recurrence rate, and length of the resected vagina and ligaments.

A study was excluded from the systematic review if it failed to report the principal demographic and clinicopathological findings of patients, including age, body mass index, International Federation of Gynecology and Obstetrics (FIGO) stage, histological findings, and tumor size.

### Data extraction

Two authors independently carried out literature searches and identified eligible articles based on the inclusion and exclusion criteria. Then each author independently extracted data from each study, including the first author, publication year, country, study design, patient characteristics, and data on the outcomes in the inclusion criteria. Discrepancies in extracted data were resolved by consensus.

### Assessment of study quality

Two authors independently assessed the quality of included studies using the guidelines in the Cochrane Handbook for Systematic Reviews of Interventions. The assessment tool contained six core items: sequence generation, allocation concealment, blinding, incomplete outcome data (e.g. about follow-up/withdrawals), selective outcome reporting and other potential sources of bias (e.g. comparability of groups). Each study was classified as having low, moderate, or high risk of bias. Discrepancies were resolved by a third author.

### Data management and statistical analysis

Data for dichotomous variables were analyzed using relative risk (RR), while data for continuous variables with the same measurement unit were analyzed using the weighted mean difference (WMD); in all cases, the binomial 95% confidence interval (95%CI) was also calculated. All statistical tests were performed using RevMan 5.2 software (Cochrane Collaboration). Possible heterogeneity among studies was evaluated using a chi squared-based Q-test or *χ* ^2^ test. Heterogeneity was also estimated using the *I* ^2^ index, which describes the percentage of total variation across studies that is due to heterogeneity rather than chance. A fixed-effects model was used if no statistical heterogeneity existed (P>0.1, *I* ^2^≤50%); otherwise, a random-effects model was used and sensitivity analysis was performed.

We planned to perform subgroup analysis in the event that we were able to identify the source of clinical heterogeneity or in the event that the included studies encompassed a range of study designs. We also planned to provide descriptive analysis of data from different studies if they could not be combined into a meta-analysis. We planned to assess publication bias by visual inspection of Begg's funnel plots if we could include a sufficient number of studies in the analysis.

## Results

### Description and quality assessment of included studies

We identified a total of 161 relevant studies in our database searches. Of these, 20 were reviews, 103 did not compare the clinical efficacy of NSRH and RH, and 11 met exclusion criteria criteria. The remaining 27 studies were read in detail. Of these, one was excluded because some patients in the LRH group underwent a nerve-sparing operation and we were unable, on the basis of the text, to separate out the data for those who received the nerve-sparing procedure and those who received the non-nerve-sparing procedure [12]. Another study was excluded because it involved robot-assisted operation [13], another because some patients underwent laparoscopic operation while others underwent laparotomy [14], and another three because cervical cancer and endometrial carcinoma patients were enrolled together [15]–[17]. A further four studies were excluded because the data could not be extracted in a form required by our software or because they did not report on at least one of the outcomes in the inclusion criteria [7], [18]–[20].

**Figure 1:**
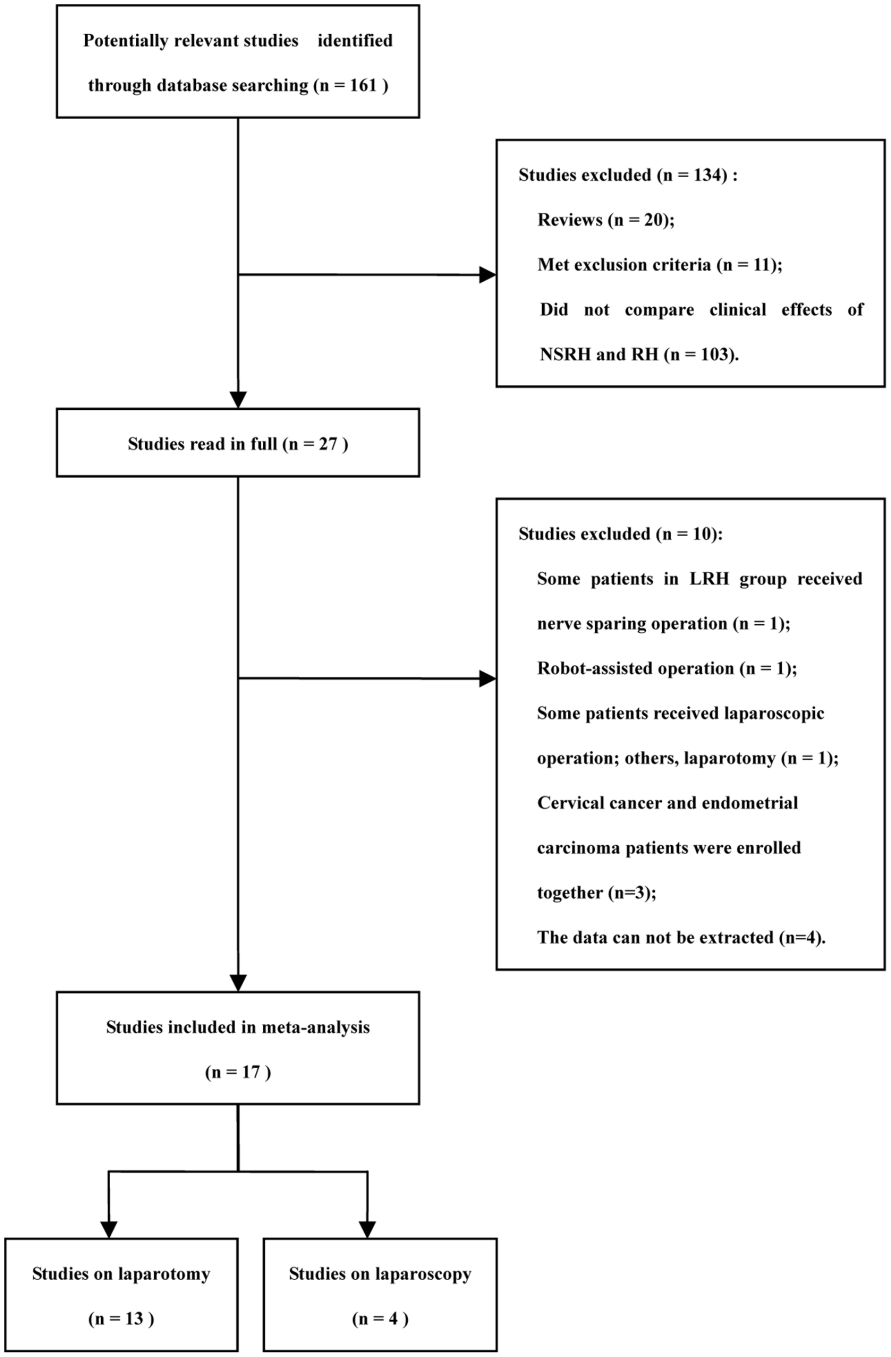
Flowchart of study selection.

In the end, 17 studies were included in the meta-analysis (Figure 1), including 13 involving laparotomy [21]–[33] and 4 involving laparoscopy [34]–[37]. Of these 17, 10 were conducted in mainland China [24], [25], [28], [29], [31], [32], [34]–[37], one in Taiwan [33], two in Japan [21], [23], two in Italy [22], [30], one in Poland [27] and one in the Netherlands [26]. One study included three groups [22], so we extracted only the data for the Type III NSRH group and the Type III RH group. Principal characteristics of all included studies are listed in Table 1.

**Table 1:** Main characteristics of all studies included in the systematic review.

Study	N		Age, yr		Body mass index, kg/m^2^		FIGO stage, n (Ia/Ib1/Ib2/IIa/IIb/III)		Histological findings, n (S/A/SA/Other types)		Tumor size	
	NSRH	RH	NSRH	RH	NSRH	RH	NSRH	RH	NSRH	RH	NSRH	RH
***Laparotomy-based approaches***												
Sakuragi 2005 [21]	22	5	43(35–60)	46 (31–64)	NR	NR	0/6/6/3/7/0	0/4/0/0/1/0	15/2/4/1	4/0/1/0/0	39 (11–70) mm	34 (12–50) mm
Raspagliesi 2006 [22]	59	20	47.3 (44.2–50.4)	47.5 (42.5–52.5)	23.9 (22.8–25.0)	25.3 (23.6–27.0)	0/32/2/7/11/1	0/12/1/1/3/2	41/13/3/2	14/3/2/1	29.3 (27.4–31.3) mm	31.4 (29.3–43.4) mm
**Todo 2006** [23]	22	5	43 (35–60)	46 (31–64)	NR	NR	0/6/6/3/7/0	0/4/0/0/1/0	NR	NR	39 (11–70) mm	34 (12–50) mm
Li 2008 [24]	22	22	42±7	44±9	23.7±3.3	23.9±2.9	0/22/14/8/0/0*		40/2/2/0*		NR	NR
Sun 2009 [25]	21	21	43±1	43±6	23.5±2.4	23.8±1.5	Ib1∼Ib2*		21/0/0/0	21/0/0/0	NR	NR
van den 2009 [26]	122	124	46.2 (23–80)	46.5 (25–81)	NR	NR	3/95/12/12/0/0	3/84/21/6/0/0	84/36/0/4	75/47/0/2	>40 mm (n = 24)	>40 mm (n = 18)
Skret 2010 [27]	10	10	48.1±12.1*		25.6±1.1*		0/10/0/0/0/0	0/8/1/1/0/0	8/1/0/1	8/2/0/0	2.6±1.1 cm	3.1±1.6 cm
Wu 2010 [28]	14	15	44.57±6.62	43.20±7.72	23.33±3.36	23.99±3.26	0/10/0/4/0/0	0/12/2/1/0/0	12/1/0/1	12/1/0/2	NR	NR
Long 2010 [29]	33	36	44.21(31–60)	45.67 (31–62)	21.5 (16.6–28.1)	22.0(17.4∼27.6)	0/15/21/6/24/0*		50/12/6/1*		NR	NR
Ditto 2011 [30]	185	311	49 (26–77)	46 (22–75)	<30 (n = 160)	<30 (n = 246)	0/20/50/34/81/0	2/173/77/34/25/0	136/29/20▴	273/23/15▴	NR	NR
					30–35 (n = 11)	30–35 (n = 27)						
					>35 (n = 2)	>35 (n = 4)						
					missing (n = 12)	missing (n = 34)						
Zhu 2011 [31]	28	33	46.5±19.2	47.5±18.3	NR	NR	0/13/5/10/0/0	0/14/10/9/0/0	22/2/3/1	26/3/4/1	≤4 cm (n = 17)	≤4 cm (n = 16)
											>4 cm (n = 11)	>4 cm (n = 17)
Chen 2012 [32]	12	13	39.5 (35–53)	45 (31–51)	21.54±2.14	22.72±2.94	0/5/2/5/0/0	0/4/4/5/0/0	NR	NR	NR	NR
Tseng 2012 [33]	18	12	42 (32–54)	45 (38–61)	NR	NR	2/28/0/0/0/0*		12/6/0/0	9/3/0/0	1.7 cm	2.8 cm
***Laparoscopy-based approaches***												
Chen 2009 [34]	37	25	44±10	45±12	NR	NR	0/8/17/12/0/0	0/7/12/16/0/0	34/3/0/0	23/2/0/0	3.2±0.8 cm	3.3±0.9 cm
Zhang 2010 [35]	17	18	41±8	39±6	24.2±3.5	24.4±3.1	3/6/5/3/0/0	2/8/4/4/0/0	17/0/0/0	18/0/0/0	NR	NR
Liang 2010 [36]	82	81	43.6±11.2	41.3±13.5	21.3±7.4	19.7±6.7	24/36/22/0/0/0	18/29/34/50/0/0	76/6/0/0	74/7/0/0	3.1±2.3 cm	3.3±2.7 cm
Lu 2012 [37]	15	15	43.0±1.0	43.0±6.2	23.5±2.2	23.7±1.8	0/12/1/2/0/0	0/9/3/3/0/0	13/1/1/0	12/1/2/0	<4 cm	<4 cm

Data are reported as mean ± SD or as median (range).

Abbreviations: A, adenocarcinoma; NR, not reported; S, squamous carcinoma; SA, adenosquamous carcinoma.

*Data for the two groups were not reported separately.

▴ Cases of SA were not reported separately from the other types of histology findings, so they have been included here as S/A/SA+Other types.

Two of the 13 studies involving laparotomy included in this meta-analysis were randomized controlled trials (RCTs) [28], [32], while 11 were non-randomized controlled trials [21]–[27], [29]–[31]. All 4 studies involving laparoscopy were non-randomized controlled trials. The risk of bias in the included studies was assessed using quality assessment tools in the Cochrane Handbook (Table 2).

**Table 2:** Quality assessment of included studies based on Cochrane Handbook core items.

Study	Randomized/Non-randomized allocation	Allocation concealment	Blinding	Follow up/withdrawal	Complete outcome reporting	Comparability
***Laparotomy-based approaches***						
***Non-randomized controlled trials***						
Sakuragi 2005 [21]	Adequate	No	Unclear	Adequate	Yes	Yes
Raspagliesi 2006 [22]	Adequate	No	Unclear	Adequate	Yes	Yes
Todo 2006 [23]	Adequate	No	Unclear	Adequate	Yes	Yes
Li 2008 [24]	Adequate	No	Unclear	Adequate	Yes	Yes
Sun 2009 [25]	Adequate	No	Unclear	Adequate	Yes	Yes
Skret 2010 [26]	Adequate	No	Unclear	Adequate	Yes	Yes
van den Tillaart 2009 [27]	Adequate	No	Unclear	Adequate (ITT)	Yes	Yes
Long 2010 [29]	Adequate	No	Unclear	Adequate	Yes	Yes
Ditto 2011 [30]	Adequate	No	Unclear	Adequate	Yes	Yes
Zhu 2011 [31]	Adequate	No	Unclear	Adequate	Yes	Yes
Tseng 2012 [33]	Adequate	No	Unclear	Adequate	Yes	Yes
***Randomized controlled trials***						
Wu 2010 [28]	Unclear	Unclear	Unclear	Adequate	Yes	Yes
Chen 2012 [32]	Unclear	Unclear	Unclear	Adequate	Yes	Yes
***Laparoscopy-based approaches***						
***Non-randomized controlled trials***						
Chen 2009 [33]	Adequate	No	Unclear	Adequate	Yes	Yes
Zhang 2010 [33]	Adequate	No	Unclear	Adequate	Yes	Yes
Liang 2010 [33]	Adequate	No	Unclear	Adequate	Yes	Yes
Lu 2012 [33]	Adequate	No	Unclear	Adequate	Yes	Yes

### Analysis of clinical efficacy and safety of laparotomy-based procedures

#### Blood loss

**Figure 2:**
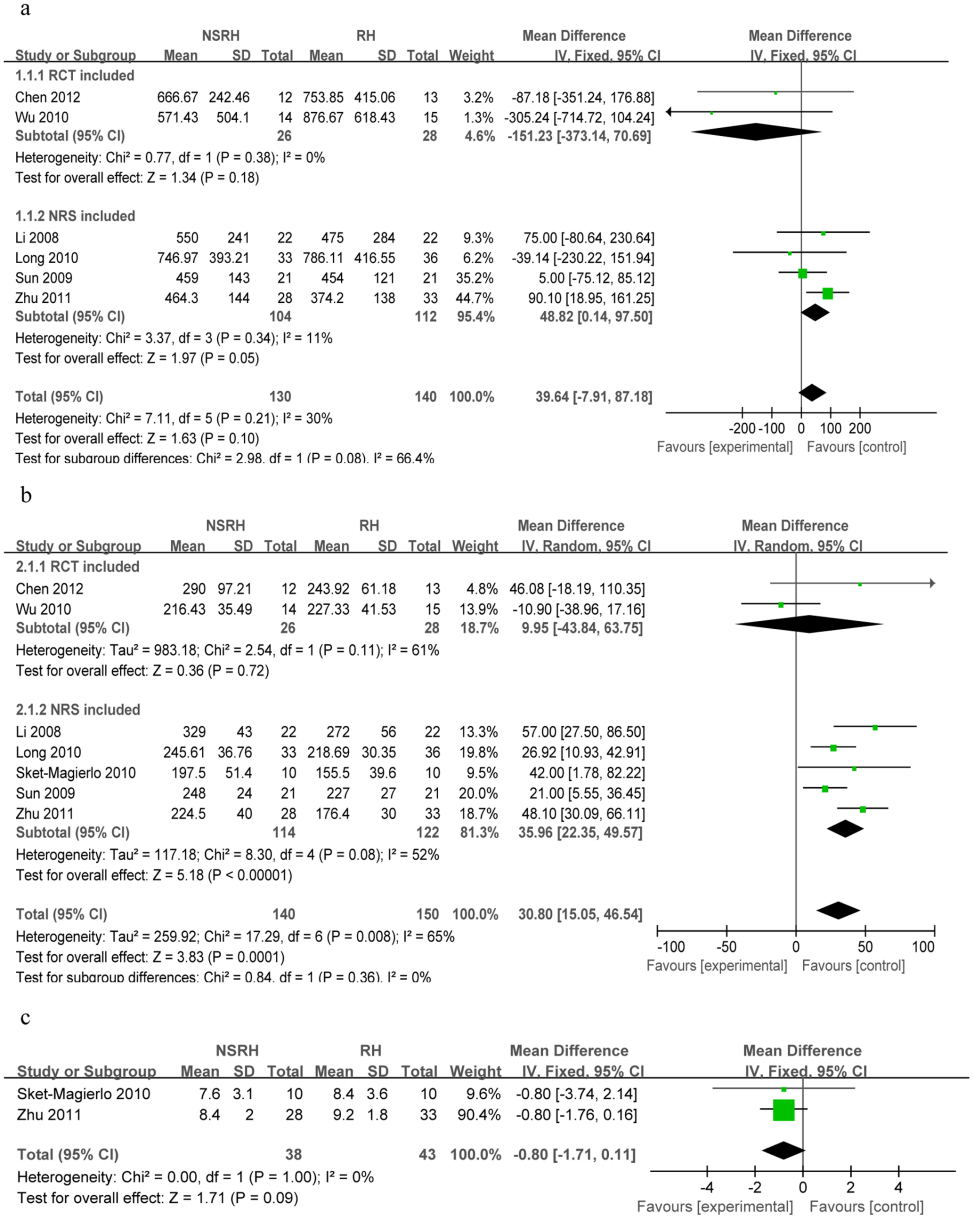
Forest plots comparing NSRH with RH in terms of (a) intraoperative blood loss, (b) operating time and (c) length of hospital stay.

While 11 studies reported data on intraoperative blood loss [21], [22], [24]–[26], [28]–[32], five could not be included in the meta-analysis because they did not report means and standard deviations [21], [22], [26], [30], [33]. The remaining six studies were divided into a subgroup comprising two RCTs [28], [32] and a subgroup of four non-randomized studies [24], [25], [29], [31]. Heterogeneity was not detected, so a fixed-effects model was used. The NSRH and RH groups showed similar blood loss within the RCT subgroup (n = 54), with a WMD of −151.23 (95%Cl −373.14 to 70.69, *P* = 0.18). Similarly, the two groups did not differ significantly among the non-randomized studies (n = 216), with a WMD of 48.82 (95%Cl 0.14 to 97.50, *P* = 0.05). The total test effect across all six RCTs and non-randomized studies was WMD = 39.64 (95%Cl −7.91 to 87.18, *P* = 0.10; Figure 2a).

#### Operating time

While 12 studies reported operating time [21], [22], [24]–[33], five could not be used in the meta-analysis because they did not report means and standard deviations [21], [22], [26], [29], [33]. The remaining seven studies were divided into a subgroup of two RCTs [28], [32] and a subgroup of five non-randomized studies [24], [25], [27], [29], [31]. Heterogeneity was detected, so a random-effects model was used. Operating time did not differ significantly between the NSRH and RH groups in the RCT subgroup (n = 54; WMD = 9.95, 95%Cl −43.84 to 63.75, *P* = 0.72), but it was significantly longer in the NSRH group among the five non-randomized studies (n = 236; WMD = 35.96, 95%Cl 22.35 to 49.57, *P*<0.00001). The total test effect across two subgroups also showed longer operating time for NSRH (WMD = 30.80, 95%Cl 15.05 to 46.54, *P* = 0.0001; Figure 2b). Sensitivity analysis showed that similar results were obtained when a fixed-effects model was used.

#### Hospital stay

A total of four studies reported data on hospital stay [27], [30], [31], [33], but two could not be used in the meta-analysis because they did not report means and standard deviations [30], [33]. Data for the remaining two studies [27], [31] were combined and meta-analyzed using a fixed-effects model because heterogeneity was not detected (*P* = 1.00, *I^2^* = 0%). The meta-analysis showed similar length of hospital stay for both the NSRH and RH groups (n = 81; WMD = −0.80, 95%Cl −1.71 to 0.11, *P* = 0.09; Figure 2c).

#### Time to recover bladder function based on post-void residual (PVR) urine volume

Of the nine studies reporting the postoperative time to recover normal post-void residual (PVR) urine volume [22], [24], [25], [27]–[29], [31]–[33], two could not be included in the meta-analysis because they did not report means and standard deviations [24], [25]. Another study was excluded because it reported data only in the form of a Kaplan-Meier curve [22], and one study was excluded because it described only the duration of spontaneous voiding [33].

Of the remaining five studies, three reported the number of postoperative days until the PVR urine volume was <50 ml [27], [29], [32].These three studies comprised one RCT [32] and a subgroup of two non-randomized studies [27], [29]. The RTC reported that average time to achieve residual urine ≤50 ml was shorter in NSRH than in RH. Meta-analysis of two non-randomized studies showed that NSRH was associated with shorter time to recover bladder function (n = 89; WMD = −5.49, 95%Cl −7.36 to −3.62, *P*<0.00001).

Three studies reported the number of postoperative days until PVR urine volume was <100 ml [28], [29], [31]; these comprised one RCT [28] and a subgroup of two non-randomized studies [29], [31]. The RCT reported that postoperative time to achieve residual urine <100 ml was much shorter in NSRH patients than in RH patients. Heterogeneity was detected, so a random-effects model was used for two non-randomized studies, which showed that NSRH was associated with shorter recovery time (n = 130; WMD = −7.36, 95%Cl −11.99 to −2.74, *P* = 0.002).

**Figure 3:**
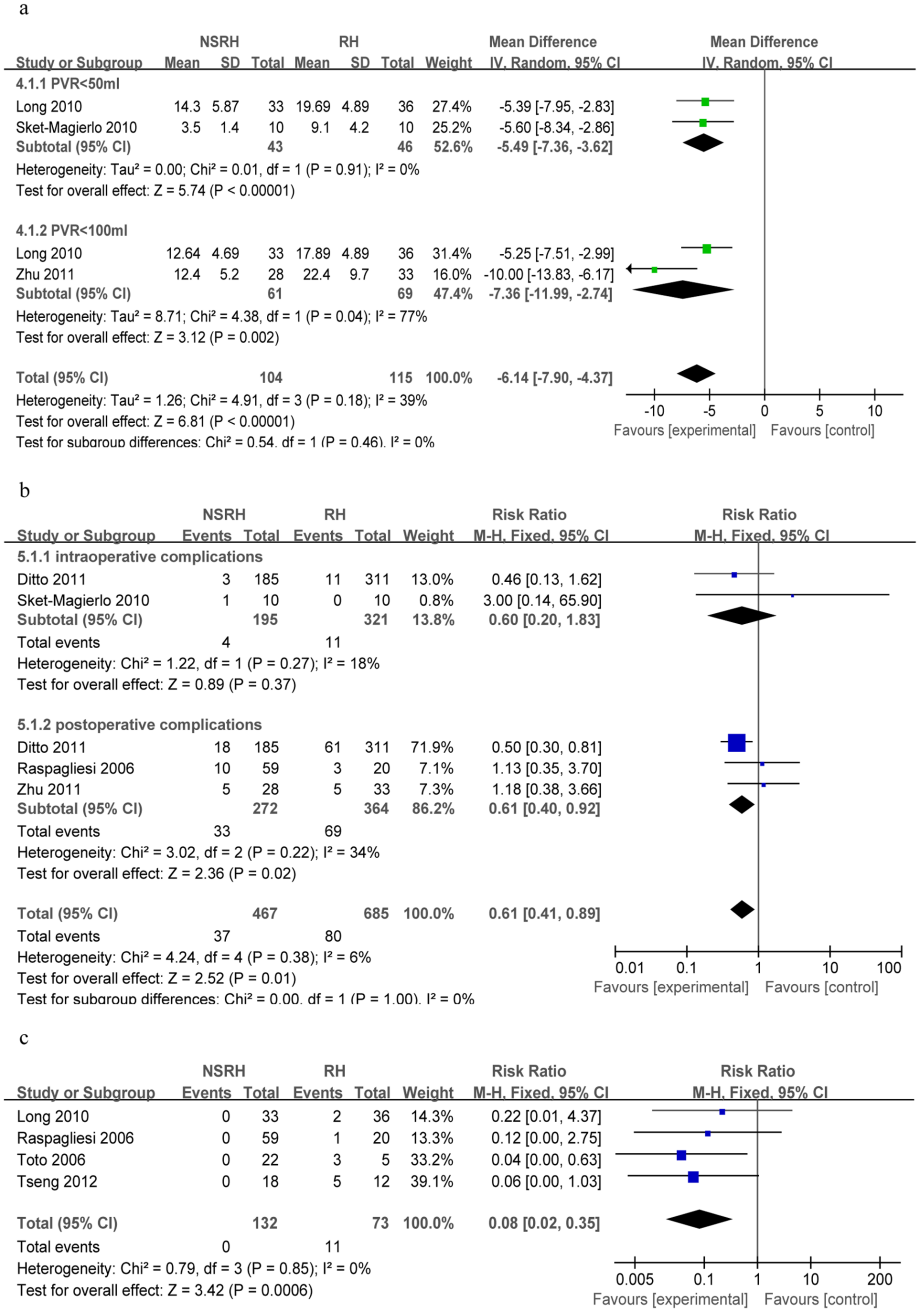
Forest plots comparing NSRH with RH in terms of (a) time to achieve normal post-void residual urine volume, (b) intra- and postoperative complications and (c) bladder dysfunction.

The total test effect for two subgroups was WMD = −6.14, 95%Cl −7.90 to −4.37 (*P*<0.00001, Figure 3a). Sensitivity analysis showed that similar results were obtained when a fixed-effects model was used.

#### Time to recover bladder function based on urodynamic study

One RCT described the results of a urodynamic study carried out 6–12 months after surgery [28]. Both the maximum flow rate (MFR) and average flow rate (AFR) were significantly better in the NSRH group (n = 7) than in the RH group (n = 7) (P<0.05). Among non-randomized studies, only one reported relevant data [23]. The NSRH group in that study showed a similar value for MFR before and 12 months after the procedure; the RH group, in contrast, showed a significant decrease in MFR over the same period. In addition, the RH group experienced much lower detrusor contraction pressure and higher abdominal pressure at maximum flow than did the NSRH group.

#### Time to recovery of anal/rectal function

Only two studies reported data for these outcomes [29], [32]. In one RCT [32], the time to first defecation was significantly shorter in the NSRH group (79.25±17.67 h) than in the RH group (99.15±23.33 h, P = 0.026). Those authors also indicated that the time to first flatus was slightly shorter in the NSRH group (50.53±14.21 h vs 62.46±18.17 h), although the difference did not achieve statistical significance (P = 0.083). One non-randomized study [29] reported that time to first flatus was significantly shorter in the NSRH group (62.99±11.99 vs 79.32±13.22 h, P<0.001), as was the time to first defecation (95.42±12.56 h vs 120.04±21.00 h, P<0.001).

#### Intra- and postoperative complications

Of the seven studies that reported data on intraoperative complications [22], [24], [25], [27], [29], [30], [33], five reported 0% incidence in both the NSRH and RH groups [22], [24], [25], [29], [33]. Of the five studies that reported data on postoperative complications [22], [24], [28], [30], [31], one RCT reported overall incidence of 28.57% (4 of 14) in the NSRH group and 53.33% (8 of 15) in the RH group [28]. In contrast, a non-randomized study reported overall incidence of 0% in both groups [24]. Data from two studies were combined for meta-analysis of intraoperative complications [27], [30], while data from three studies were combined for meta-analysis of postoperative complications [22], [30], [31]. A fixed-effects model was used because no heterogeneity was detected. While the two techniques were associated with similar risk of intraoperative complications (n = 561; RR = 0.60, 95%Cl 0.20 to 1.83, *P* = 0.37), NSRH was associated with lower risk of postoperative complications (n = 636; RR = 0.61, 95%Cl 0.40 to 0.92, *P* = 0.02; Figure 3b).

#### Bladder dysfunction

Six studies reported data on urinary incontinence [21]–[23], [28], [29], [33]. Two studies involved the same cohort of patients, so relevant data were taken from only one of them [21], [23]. In one RCT [28], no patients in the NSRH group experienced this complication, while 2 of 15 patients (13.33%) in the RH group did. Data for the remaining studies were meta-analyzed using a fixed-effects model because no heterogeneity was detected [22], [23], [29], [33]. This analysis showed that NSRH was associated with lower risk of urinary incontinence (n = 205; RR = 0.08, 95%Cl 0.02 to 0.35, *P* = 0.0006; Figure 3c).

Abnormal bladder sensation was reported in two studies involving the same cohort of patients [21], [23], so meta-analysis was not used. Two of 22 patients (9.1%) in the NSRH group experienced this complication, compared to 3 of 5 patients (60%) in the RH group.

Another study reported data on urinary complications at six months after the operation [33]; these included nocturia, excessively urgent and frequent urination, postoperative urine retention, dysuria, and voiding difficulty. Unfortunately, the total urological incidence data were not reported in sufficient detail, so meta-analysis was not performed.

Overall, the data from these studies reporting on abnormal bladder sensation and urinary complications indicated a lower rate of bladder dysfunction in the NSRH group than in the RH group.

#### Cervical cancer recurrence rate

One study [23] found similar 4-year recurrence rates in the NSRH and RH groups, while another found similar 2-year recurrence rates [26]. In one study with a follow-up time of 14 months [27] and another in which the range of follow-up was 26–37 months [32], no cases of relapse or metastasis were reported. In addition, one study reported 30 relapses among 185 patients (16.22%) in the NSRH group after 42 months of follow-up and 60 relapses among 311 patients (19.29%) in the RH group after 159 months of follow-up [30].

#### Survival rate

One study reported similar 5-year disease-free survival (DFS) for NSRH (78.9%) and RH (79.8%; P = 0.519), and similar 5-year overall survival (OS) (90.8% in NSRH vs 84.1% in RH, P = 0.192) [30]. Another study reported similar 5-year overall OS curves for NSRH and RH groups [26]. One study reported that all cases were free of disease after a median follow-up of 12 months (range, 9–16 months) [33]. Another study reported similar duration of DFS after 48 months in the two groups [23]. Although these studies varied in follow-up time and some did not report individual survival times and so could not be combined in a meta-analysis, they consistently showed similar survival rates for NSRH and RH.

#### Extent of resection

One RCT reported similar cardinal ligament lengths in the NSRH group (37.2±7.7 mm; range, 30.0–55.0) and RH group (36.8±5.3 mm; range, 30.0–50.0 mm) [32]. Two non-randomized studies reported similar parametrial widths and vaginal cuff lengths for both groups [21], [29], although one study reported only median and range values [21]. In all three studies, the extent of resection was similar in the two groups.

### Analysis of clinical efficacy and safety of laparoscopy-based procedures

#### Blood loss

**Figure 4:**
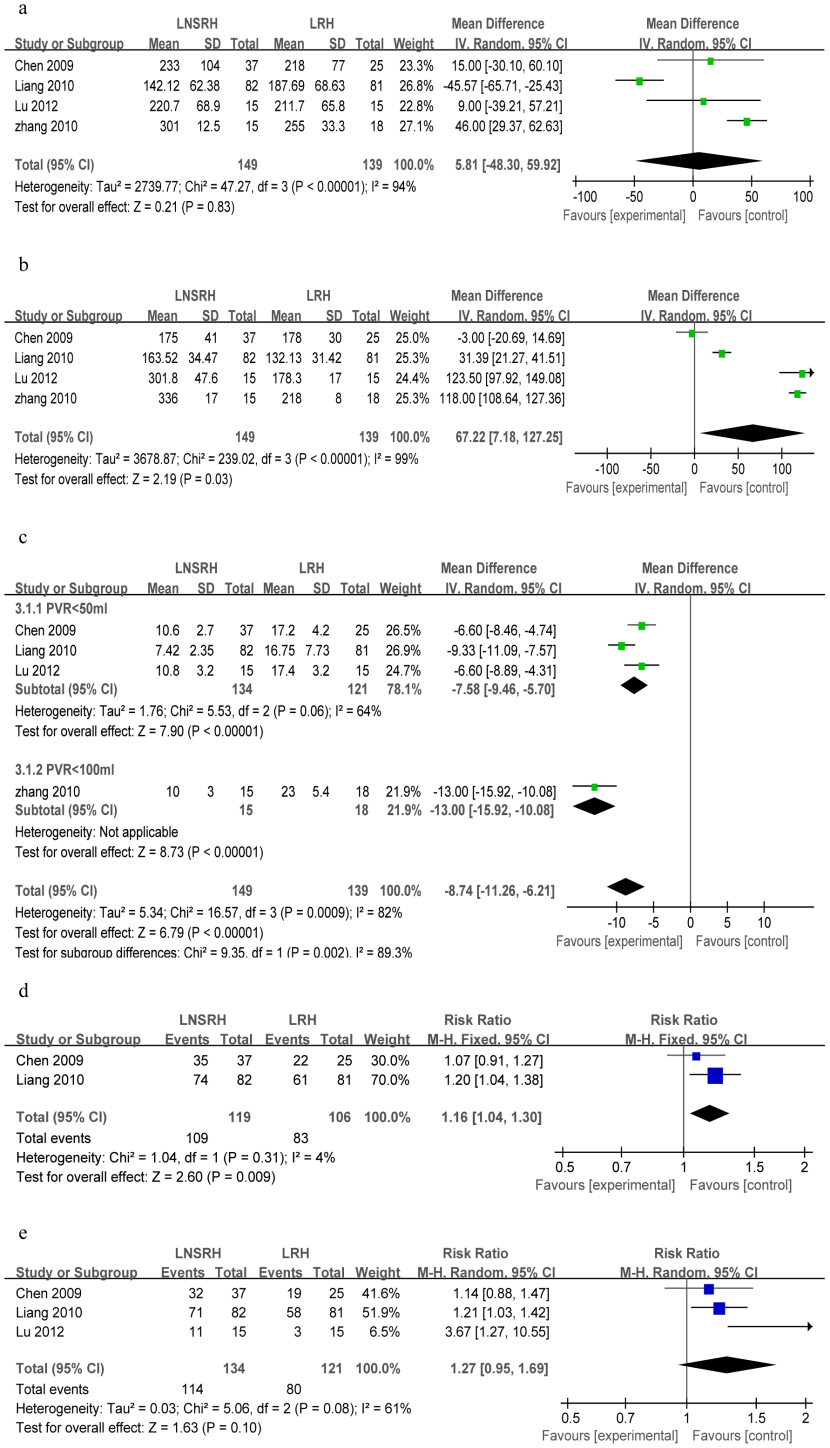
Forest plots comparing LNSRH with LRH in terms of (a) intraoperative blood loss, (b) operating time, (c) time to recover bladder function based on post-void residual (PVR) urine volume, (d) bladder function recovery based on postoperative sensation of bladder fullness and (e) bladder function recovery based on postoperative satisfaction with micturition.

Four laparoscopic studies reported data on intraoperative blood loss [34]–[37]. Heterogeneity was detected, so a random-effects model was used. The LNSRH and LRH groups showed similar blood loss (n = 288), with a WMD of 5.81 (95%Cl −48.30 to 59.92, *P* = 0.83; Figure 4a). Sensitivity analysis showed that similar results were obtained when a fixed-effects model was used.

#### Operating time

Four non-randomized laparoscopic studies reported operating time [34]–[37]. Heterogeneity was detected, so a random-effects model was used. Operating time was significantly longer in the LNSRH group than in the LRH group (n = 288; WMD = 67.22, 95%Cl 7.18 to 127.25, *P* = 0.03; Figure 4b). Sensitivity analysis showed that similar results were obtained when a fixed-effects model was used.

#### Hospital stay

Only one study reported data on hospital stay [37]. The authors reported shorter length of hospital stay in the LNSRH group (10.9±2.0 d; n = 15) than in the LRH group (15.1±0.8 d; n = 15; *P*<0.05).

### Time to recover bladder function based on post-void residual (PVR) urine volume

Four studies reported the postoperative time to recover normal post-void residual (PVR) urine volume [34]–[37]. They comprised one subgroup of three studies reporting the number of postoperative days until the PVR urine volume was ≤50 ml [34]–[37] and one subgroup of a single study reporting the number of postoperative days until the PVR urine volume was ≤100 ml [35]. Meta-analysis showed that LNSRH groups was associated with shorter average time to achieve PVR urine volume ≤50 ml (n = 256; WMD = −7.58, 95%Cl −9.46 to −5.70, *P*<0.00001) or ≤100 ml (n = 33; WMD = −13.00, 95%Cl −15.92 to −10.08, *P*<0.00001; Figure 4c).

Meta-analysis of the two subgroups together showed shorter recovery time for LNSRH: WMD = −8.74, 95%Cl −11.26 to −6.21 (*P*<0.00001, Figure 4c). Sensitivity analysis showed that similar results were obtained when a fixed-effects model was used.

#### Bladder function recovery based on postoperative sensation of bladder fullness and satisfaction with micturition

Two studies described the sensation of bladder fullness in patients after surgery [34], [36], which we meta-analyzed using a fixed-effects model. The significant difference was found between the LNSRH and LRH groups (WMD = 1.16, 95%Cl 1.04 to 1.30, *P* = 0.009, Figure 4d). Three studies described patient satisfaction with micturition [34], [36], [37]. Heterogeneity was detected, so a random-effects model was used. Meta-analysis showed a similar result for the two groups (WMD = 1.27, 95%Cl 0.95 to 1.69, *P* = 0.10, Figure 4e).

#### Bladder function recovery based on postoperative grade of bladder function

**Figure 5:**
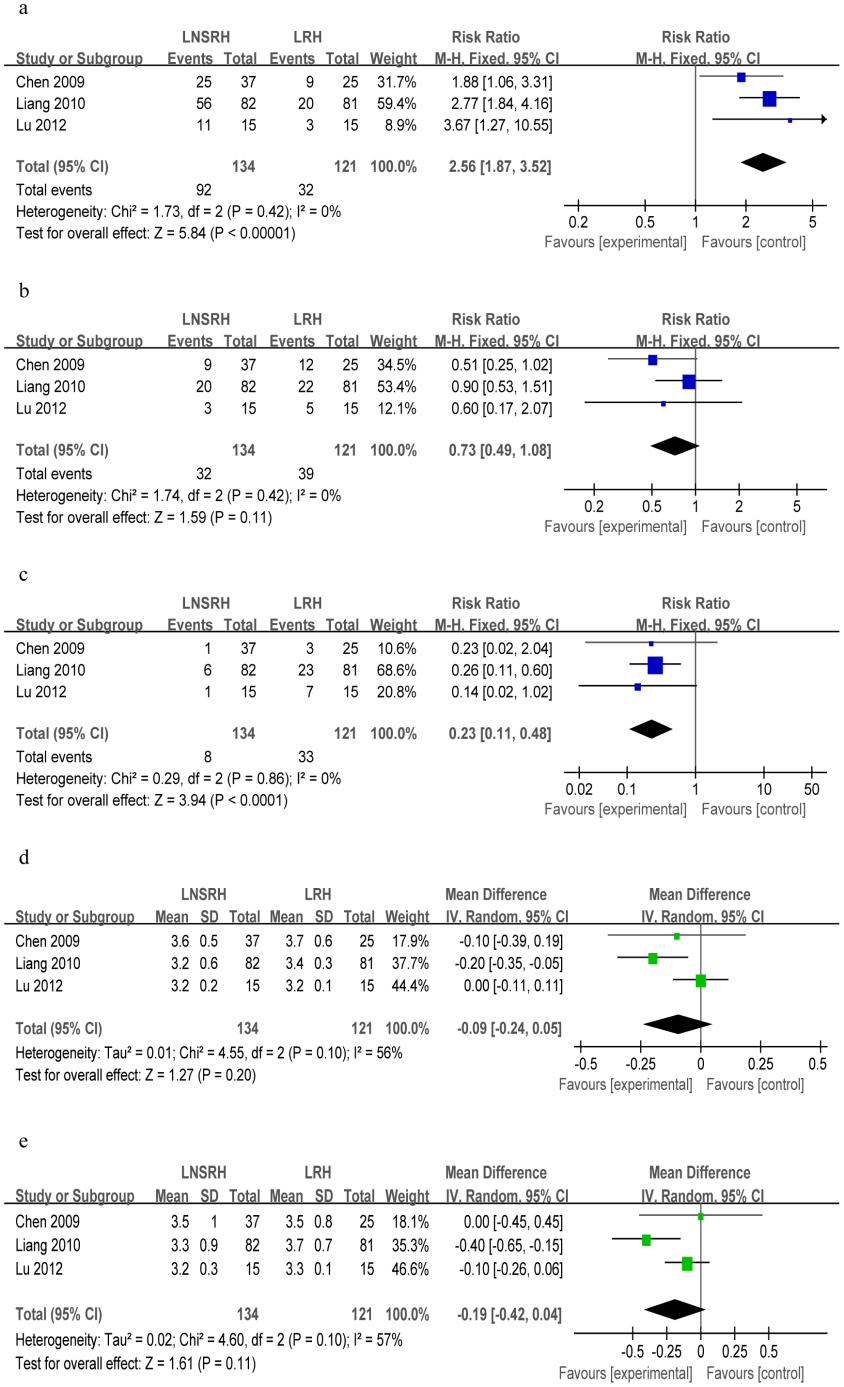
Forest plots comparing LNSRH with LRH in terms of bladder function recovery based on different grades of postoperative bladder function: (a) grade 0, (b) grade I, (c) grade II. The remaining Forest plots compare the two techniques in terms of (d) resectable parametrial width and (e) vaginal cuff length.

Three studies described the grading of bladder function after surgery [34], [36], [37]. Meta-analysis showed that LNSRH was associated with a significantly higher rate of recovery to Grade 0 than was LRH (n = 255; WMD = 2.56, 95%Cl 1.87 to 3.52, *P*<0.00001; Figure 5a), but a lower rate of Grade II (WMD = 0.23, 95%Cl 0.11 to 0.48, *P*<0.0001; Figure 5c). Both techniques, however, were associated with similar rates of recovery to Grade I (WMD = 0.73, 95%Cl 0.49 to 1.08, *P* = 0.11; Figure 5b).

#### Time to recovery of anal/rectal function

Only one study reported data for these outcomes [37]. The time to first flatus was significantly shorter in the LNSRH group (2.2±0.6 d) than in the LRH group (2.3±0.4 d, *P*<0.05).

#### Intra- and postoperative complications

Of the three studies reporting relevant data, one reported 0% incidence of intra-operative complications in both the LNSRH and LRH group [34]; another reported that only one of 81 patients in the LRH group and none of the 82 patients in the LNSRH group experienced intra-operative complications [36]. The third study reported postoperative incidence of lymphocysts: 4 cases (26.7%) in the LNSRH group and 5 cases (33.3%) in the LNSRH group [37]; these two rates were not significantly different (P>0.05).

#### Cervical cancer recurrence rate

No cases of relapse or metastasis were reported in one study involving follow-up of 11–19 months [34] or in two studies in which follow-up ranged from 5 to 42 months [36] or from 3 to 19 months [37].

#### Extent of resection

Three studies reported resectable parametrial widths and vaginal cuff lengths [34], [36], [37]. Heterogeneity was detected, so a random-effects model was used to meta-analyze the data. The results showed similar extent of resection between the two groups for vaginal cuff length (n = 255; WMD = −0.19, 95%Cl −0.42 to 0.04, *P* = 0.11; Figure 5d) and for parametrial width (WMD = −0.09, 95%Cl −0.24 to 0.05, *P* = 0.20; Figure 5e). Sensitivity analysis showed that similar results were obtained when a fixed-effects model was used.

### Assessment of publication bias

Since we could not include a sufficient number of studies in the outcomes meta- analysis, we did not assess publication bias by visual inspection of Begg's funnel plots.

## Discussion

NSRH and LNSRH have become increasingly common in clinical practice, in large part because the procedure is thought to protect the autonomic pelvic plexus during surgery and thereby reduce postoperative morbidity compared to conventional RH and LRH. In order to examine whether this belief is well-founded, we performed a systematic review of the literature comparing the clinical efficacy and complications of NSRH and RH based on laparotomy or laparoscopy. Our findings support the results of individual studies indicating that NSRH leads to more rapid recovery of bladder function and decreases incidence of bladder dysfunction [8], [18], [38], [39]. We also found NSRH to be associated with lower risk of postoperative complications. These conclusions are consistent with at least two studies that we were unable to include in the meta-analysis because they examined only LNSRH but not LRH. Park NY et al reported the return rates to normal voiding function for LNSRH at postoperative 14 and 21 days were 92.0% and 95.2%, respectively [40]. Putambeker SP et al reported The median return time for normal bladder function was 2 days and none of the patients reuired catheterization beyond 2 weeks [9].

The two approaches were associated with similar rates of intraoperative complications, such as bladder injury, fistula/ureter injury, thromboembolism, and blood transfusion [27], [30], [33]. They were also associated with similar amounts of intraoperative blood loss and length of hospital stay, although one laparoscopic study reported shorter length of hospital stay with the nerve-sparing procedure [37]. Meta analysis involving only non-randomized studies showed abdominal and laparoscopic NSRH to be associated with longer operating time than the corresponding RH procedures.

NSRH is thought to be associated with better postoperative anorectal and sexual function, yet we found little relevant data in the studies included in this systematic review. One RCT [32] and two non-randomized trails [29], [37] reported that NSRH is associated with faster recovery of anorectal function than is RH; unfortunately, we could not meta-analyze the data because the laparotomy trials differed in design and because there was only one laparoscopy trial. Therefore this finding should be confirmed in large, randomized clinical trials. We identified one study [41] that examined vaginal blood flow during sexual stimulation in patients treated with NSRH or RH; NSRH was associated with better overall vaginal blood flow and less denervation of the vagina. This study used photoplethysmography to measure vaginal pulse amplitude, which has proven to be a reliable index of vaginal vasocongestion [42].

The results of our systematic analysis and meta-analysis suggest that NSRH is associated with fewer complications and faster recovery of certain functional outcomes than is RH. We also wanted to compare the oncological efficacy of the two surgical approaches. Sakuragi et al. reported cumulative DFS rates of 95.5% for NSRH and 100% for RH at 24 months [21], while van den Tillaart et al. observed similar 5-year overall OS and local recurrence rates within 24 months for NSRH and RH [26]. The finding that NSRH and RH are associated with similar survival may reflect our meta-analysis findings that they are associated with a similar extent of resection, based on three abdominal and three laparoscopic studies of parametrial width and vaginal cuff length. This is contrary to the belief among some clinicians that NSRH involves less extensive resection and therefore can lead to lower survival and higher risk of recurrence. Our findings on survival and recurrence should be interpreted with caution because they come from individual studies of relatively limited statistical power, which could not be combined because of differences in study design and outcomes reporting.

Our finding that NSRH and RH are associated with similar survival is supported by at least one study that we were unable to include in our meta-analysis: in an uncontrolled study involving only LNSRH, Putambeker et al reported 5-year DFS rates of 92% for IB1 cervical cancer and 78% for IB2 cervival cancer, with corresponding 5-year OS rates of 96% and 83% [9]. The authors concluded that LNSRH does not compromise surgical radicality.

Faced with a diversity of NSRH techniques, such as laparotomy, laparoscopic operation, and robot-assisted operation [13], [43]–[45], we decided to include data based on NSRH and RH treated by laparotomy or laparoscopy. We meta-analyzed the two approaches separately, given that laparoscopic surgery is more difficult than laparotomy, it requires more time and a different skill set, and it is often applied to patients with substantially different profiles than those in whom laparotomy is performed [36], [46], [47]. Thus combining outcomes from both laparoscopic surgery and laparotomy in the same meta-analysis would likely increase clinical heterogeneity and render the results less reliable.

Many of the conclusions of this systematic review are based on individual studies because the data could not be pooled for meta-analysis. Some results are based on only two studies, raising significant issues of statistical power. Therefore our findings should be verified in larger, controlled trials that report more extensive data on clinical efficacy and safety and that cover more of the NSRH approaches currently used in the clinic.

Our findings are also subject to various types of bias. One is selection bias: we had to include both randomized and non-randomized trials given that we were able to include only two eligible RCTs [28], [32]. Our results may have been different if we had been able to include enough RCTs to omit the non-randomized studies. Our findings may also reflect publication bias: we did not include articles written in languages other than English or Chinese, nor did we include unpublished data.

In conclusion, this systematic review and meta-analysis provides evidence for the belief that NSRH is associated with low postoperative morbidity and good clinical efficacy for treating patients with early cervical cancer, which has helped make it an increasingly popular clinical option. It appears to be as safe as traditional RH in terms of intraoperative blood loss, length of hospital stay, long-term survival and recurrence rate, although it is associated with longer operating time. Many of these findings are based on a relatively small number of trials, most of which were non-randomized and could not be pooled for meta-analysis. Therefore they should be verified in larger, multi-center RCTs.

## Supporting Information

Checklist S1 **PRISMA checklist.**
(DOC)
