# Correction: Clinical Efficacy and Safety of Nerve-Sparing Radical Hysterectomy for Cervical Cancer: A Systematic Review and Meta-Analysis

**DOI:** 10.1371/journal.pone.0101068

**Published:** 2014-06-24

**Authors:** The PLOS ONE Staff

## Notice of Republication

This article was republished on May 27, 2014, to include Checklist S1, the PRISMA checklist, which was mistakenly omitted during the production process. The publisher apologizes for the error. Please download this article again to view the correct version. The originally published, uncorrected article and the republished, corrected article are provided here for reference.

## Supporting Information

File S1 Originally published, uncorrected article.
(PDF)

File S2 Republished, corrected article.
(PDF)
